# Factors influencing serum neurofilament light chain levels in normal aging

**DOI:** 10.18632/aging.203790

**Published:** 2021-12-18

**Authors:** Marisa Koini, Lukas Pirpamer, Edith Hofer, Arabella Buchmann, Daniela Pinter, Stefan Ropele, Christian Enzinger, Pascal Benkert, David Leppert, Jens Kuhle, Reinhold Schmidt, Michael Khalil

**Affiliations:** 1 Clinical Division of Neurogeriatrics, Department of Neurology, Medical University of Graz, Graz, Austria; 2 Institute for Medical Informatics, Statistics and Documentation, Medical University of Graz, Graz, Austria; 3 Department of Neurology, Medical University of Graz, Graz, Austria; 4 Division of Neuroradiology, Vascular and Interventional Radiology, Department of Radiology, Medical University of Graz, Graz, Austria; 5 Neurology Clinic and Policlinic, MS Center and Research Center for Clinical Neuroimmunology and Neuroscience Basel (RC2NB), University Hospital Basel, University of Basel, Basel, Switzerland

**Keywords:** serum neurofilament light (sNfL), confounders, age, BMI, renal function

## Abstract

Objective: Serum neurofilament light (sNfL) is a promising marker for neuro-axonal damage and it is now well known that its levels also increase with higher age. However, the effect of other determinants besides age is still poorly investigated. We therefore aimed to identify factors influencing the sNfL concentration by analysing a large set of demographical, life-style and clinical variables in a normal aging cohort.

Methods: sNfL was quantified by single molecule array (Simoa) assay in 327 neurologically inconspicuous individuals (median age 67.8±10.7 years, 192 female) who participated in the Austrian Stroke Prevention Family Study (ASPS-Fam). Random forest regression analysis was used to rank the association of included variables with sNfL in the entire cohort and in age-stratified subgroups. Linear regression then served to identify factors independently influencing sNfL concentration.

Results: Age (β=0.513, p<0.001) was by far the most important factor influencing sNfL, which was mainly driven by individuals ≥60 years. In age stratified sub-groups, body mass index (BMI) (β=-0.298, p<0.001) independently predicted sNfL in individuals aged 38-60 years. In individuals ≥60 years, age (β=0.394, p<0.001), renal function (β=0.376, p<0.001), blood volume (β=-0.198, p=0.008) and high density lipoprotein (HDL) (β=0.149, p=0.013) were associated with sNfL levels.

Conclusions: Age is the most important factor influencing sNfL concentrations, getting increasingly relevant in elderly people. BMI further influences sNfL levels, especially at younger age, whereas renal function gets increasingly relevant in the elderly.

## INTRODUCTION

In the last years, the neurofilament light (NfL) chain protein has evolved into the most promising biomarker to indicate neuro-axonal damage being at the doorstep of clinical application. Neurofilaments are scaffolding cytoskeletal proteins exclusively expressed within neurons [1]. They belong to a family of neuronal intermediate filaments that are involved in axonal growth and stability, through incorporation into different supramolecular assemblies, also in synaptic organization and function in the CNS [1, 2]. Neurofilaments can be distinguished based on their molecular masses. The largest one is neurofilament heavy chain (NfH), followed by the medium chain (NfM), the light chain (NfL), a-internexin and peripherin [1, 2]. Rising levels of neurofilaments can be found as a consequence of neuro-axonal damage and their levels are now routinely being measured using ultra-sensitive immunoassays. Currently, most evidence exists in particular for NfL as a biochemical marker for neuro-axonal damage. The mechanisms and kinetics of neurofilament protein release from neurons and trafficking between brain and blood compartment is yet still not completely clear [2]. With the advent of a single-molecule array (SiMoA) technology, reliable quantification of low NfL concentrations in the peripheral blood compared to cerebrospinal fluid (CSF) became available [3]. This cutting-edge technique facilitated to comprehensively study this protein as diagnostic and prognostic marker in various acute and chronic neurological disorders [1]. It is now also well known that NfL increases in normal aging, which is paralleled by a higher variability in the elderly [4, 5], making it difficult to correctly interpret this marker on an individual level. It is further not completely clear if other factors besides age may impact NfL levels, and if this may vary across different age ranges. There is some evidence that blood NfL may be influenced by body mass index (BMI) [6, 7], renal function [8] and blood volume [6]. However, in order to use this marker in the clinical setting, it is inevitable to exactly elucidate if and to which extent other factors besides age influence NfL levels. We therefore aimed to comprehensively study the influence and relative magnitude of various demographical, life-style and clinical factors on sNfL across different age ranges in a normal aging cohort. We hypothesized that factors which confound sNfL as well as their relative impact on this marker may change during the course of aging.

## RESULTS

### Study cohort

The entire cohort consisted of 327 neurologically inconspicuous community-dwelling participants. Table 1 displays demographical and life-style related factors, renal and liver function, cerebrovascular risk factors and blood volume for the entire cohort and in age-stratified subsamples <60 years and ≥60 years.

**Table 1:** Demographical factors and determinants of sNfL.

	**Total (n=327)** **mean (SD)**	**<60 years (n=99)** **mean (SD)**	**≥60 years (n=228)** **mean (SD)**
*demographical factors*			
age, years	64.6 (10.7)	50.1 (4.5)	70.9 (4.9)
sex, female [%]	192 [58.7]	54 [54.5]	138 [60.5]
*life-style related factors*			
waist-hip-ratio	0.9 (0.1)	0.9 (0.1)	0.9 (0.1)
BMI	26.6 (4.7)	25.8 (4.8)	26.9 (4.7)
alcohol, yes [%]	196 [59.9]	64 [64.6]	132 [57.9]
*kidney function*			
eGFR	74.2 (14.9)	84.6 (11.8)	69.7 (13.8)
creatinine [mg/dl]	0.9 (0.2)	0.9 (0.2)	1 (0.2)
Urea [mg/dl]	33.9 (11.1)	29.6 (7.9)	35.8 (11.7)
uric acid [mg/dl]	5.5 (1.4)	5.3 (1.3)	5.6 (1.4)
*liver function*			
GPT [U/l]	24.3 (11.8)	26.6 (13.5)	23.3 (10.9)
GOT [U/l]	26.1 (7.8)	25.7 (8.8)	26.3 (7.3)
GGT [U/l]	31.4 (27.6)	33.5 (38.6)	30.5 (21.2)
AP [U/l]	66.3 (17.4)	63.4 (17.9)	67.5 (17.1)
*cerebrovascular risk factors*			
hypertension, yes [%]	228 [69.7]	47 [47.5]	181 [79.4]
hypercholesterolemia, yes [%]	54 [16.5]	2 [2]	52 [22.8]
diabetes, yes [%]	24 [7.3]	1 [1]	23 [10.1]
systolic blood pressure [mmHg]	137.9 (21.5)	127.1 (18.8)	142.6 (21)
diastolic blood pressure [mmHg]	86.7 (9.3)	87 (9.9)	86.6 (9)
Cholesterol [mg/dl]	209.2 (39.5)	208.9 (35)	209.4 (41.3)
HDL [mg/dl]	68.4 (20.1)	67.6 (21.5)	68.7 (19.5)
LDL [mg/dl]	119.6 (31.9)	118.3 (29.4)	120.2 (33)
Triglycerides [mg/dl]	116.9 (72.4)	123.6 (94.8)	114 (60.2)
Hba1c [mg/dl]	5.6 (0.5)	5.4 (0.3)	5.7 (0.6)
*blood volume*	4.5 (0.8)	4.7 (0.9)	4.4 (0.7)

Mean and standard deviation (SD) are shown if not otherwise stated.

### Identification of factors influencing sNfL

When considering the entire cohort we found significant correlations of sNfL with age (rho=0.732, p<0.001), renal function (estimated glomerular filtration rate (eGFR) (rho=-0.523, p<0.001), creatinine (rho=0.152, p=0.006) and urea (rho=0.294, p<0.001)), liver function (glutamate pyruvate transaminase (GPT) (rho=-0.171, p=0.002)), cerebrovascular risk factors (systolic blood pressure (rho=0.254, p<0.001), HDL (rho=0.130, p=0.019) and Hba1c (rho=0.255, p<0.001)) and blood volume (rho=-0.240, p<0.001). Although, overall BMI was unrelated to sNfL in the total cohort, we found more indication of a correlation between sNfL and BMI in younger individuals (<60 years: rho=-0.283, p=0.005) compared participants aged 60 years and older (≥60 years: rho=-0.148, p=0.02).

Analyses on group differences revealed higher sNfL concentrations in hypertensive individuals compared to normo-tensive individuals (U=8455.0, p<0.001, sNfL-mean_hypertension_=37.0 (SD=16.2) vs. sNfL-mean_no-hypertension_=30.2 (SD=14.9)), individuals with hypercholesterolemia (U=4700.5, p<0.001, sNfL-mean_hyperchol_=43.3 (SD=16.1) vs. sNfL-mean_no-hyperchol_=33.3 (SD=15.6)) and diabetic individuals (U=2678.5, p=0.026, sNfL-mean_diabetic_=42.8 (SD=18.7) vs. sNfL-mean_non-diabetic_=34.3 (SD=15.7)).

The above-mentioned variables significantly associated with sNfL were used for further analyses.

### Conditional importance of factors influencing sNfL

We then performed random forest regressions to determine the conditional importance of each factor influencing sNfL.

**Figure 1:**
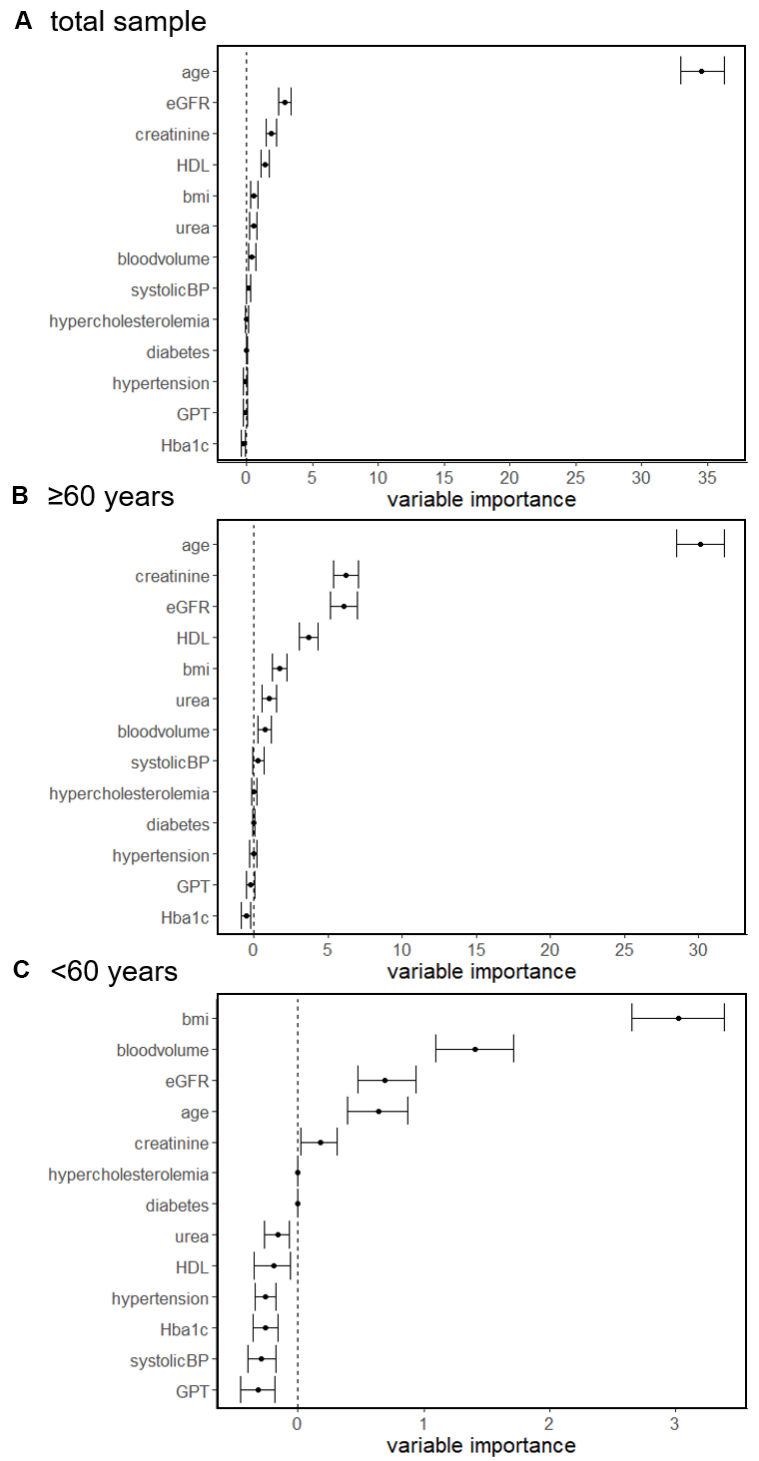
Random forest regressions of determinants associated with sNfL concentration in the total sample (**A**), individuals ≥60 years (**B**) and <60 years (**C**).

When considering the entire cohort, random forest regression revealed that among all examined factors age (mean=34.5, CI: 32.9, 36.3) had by far the highest conditional variable importance influencing sNfL, which was followed by eGFR (mean=2.9, CI: 2.4, 3.4) and creatinine (mean=1.9, CI: 1.5, 2.3) (Figure 1A).

Similar results were found in a subgroup analysis when analyzing individuals ≥60 years. In this subgroup age (mean=30.1, CI: 28.5, 31.7) had the highest conditional importance which was followed by creatinine (mean=6.2, CI: 5.4, 7.1), eGFR (mean=6.0, CI: 5.1, 7.0) and HDL (mean=3.7, CI: 3.0, 4.3) (Figure 1B).

In contrast, when analyzing in individuals <60 years we found that BMI (mean=3.0, CI: 2.7, 3.4), blood volume (mean=1.4, CI: 1.1, 1.7) and eGFR (mean=0.7, CI: 0.5, 0.9) had the highest conditional importance, whereas the impact of age (mean=0.6, CI: 0.4, 0.9) was lower (Figure 1C).

### Significant association of identified factors influencing sNfL

Multiple regression analyses then served to determine statistically significant associations of factors with highest conditional importance from random forest analyses influencing sNfL.

Considering all individuals, multiple regression analysis revealed that age (β=0.513, 95% CI: 0.631, 0.919, p<0.001) was the most important factor influencing sNfL, which was followed by eGFR (β=-0.207; 95% CI: -0.355, -0.1; p<0.001), BMI (β=-0.165; 95% CI: -0.841, -0.294; p=0.001) and urea (β=0.113; 95% CI: 0.005, 0.322; p=0.019). Overall, this model explained 49.5% of the variance (Table 2).

**Table 2:** Bootstrapped linear regression.

**Determinant**	**b^a,b^ [CI]**	**SE_b_^a^**	**Beta**	**t**	**p**	**R^2^**
*Entire sample*						
**age**	0.771 [0.631 - 0.919]	0.073	0.513	10.525	**<0.001**	0.495
**eGFR**	-0.223 [-0.355 - (-0.1)]	0.060	-0.207	-4.033	**<0.001**	
HDL	0.049 [-0.029 - 0.127]	0.040	0.061	1.358	0.175	
**BMI**	-0.559 [-0.841 - (-0.294)]	0.144	-0.165	-3.441	**0.001**	
**urea**	0.164 [0.005 - 0.322]	0.081	0.113	2.362	**0.019**	
blood volume	-0.104 [-1.943 - 1.832]	0.939	-0.005	-0.104	0.918	
<60 years						
**BMI**	-0.413 [-0.719 - (-0.144)]	0.147	-0.298	-2.519	**0.013**	0.17
blood volume	-0.478 [-2.21 - 1.206]	0.865	-0.065	-0.527	0.600	
eGFR	-0.084 [-0.184 - 0.017]	0.050	-0.149	-1.546	0.126	
age	0.182 [-0.07 - 0.462]	0.128	0.123	1.230	0.222	
≥60 years						
**age**	1.24 [0.868 - 1.632]	0.192	0.394	7.089	**<0.001**	0.379
**creatinine**	25.183 [13.912 - 35.866]	6.088	0.376	5.041	**<0.001**	
**HDL**	0.118 [0.01 - 0.215]	0.052	0.149	2.510	**0.013**	
BMI	-0.256 [-0.643 - 0.112]	0.195	-0.077	-1.163	0.246	
urea	0.072 [-0.12 - 0.275]	0.097	0.055	0.782	0.435	
**blood volume**	-4.092 [-6.797 - (-1.048)]	1.418	-0.198	-2.671	**0.008**	

^a^confidence interval and standard error with BCa-Bootstrapping with 10.000 BCa-samples.

^b^confidence intervals were used to obtain significance.

Regression coefficients, confidence intervals and standard errors are from bootstrapping. Note: variables violating multicollinearity (i.e. eGFR and creatinine) leaded to the exclusion of the variable with higher variance inflation factor.

**Figure 2:**
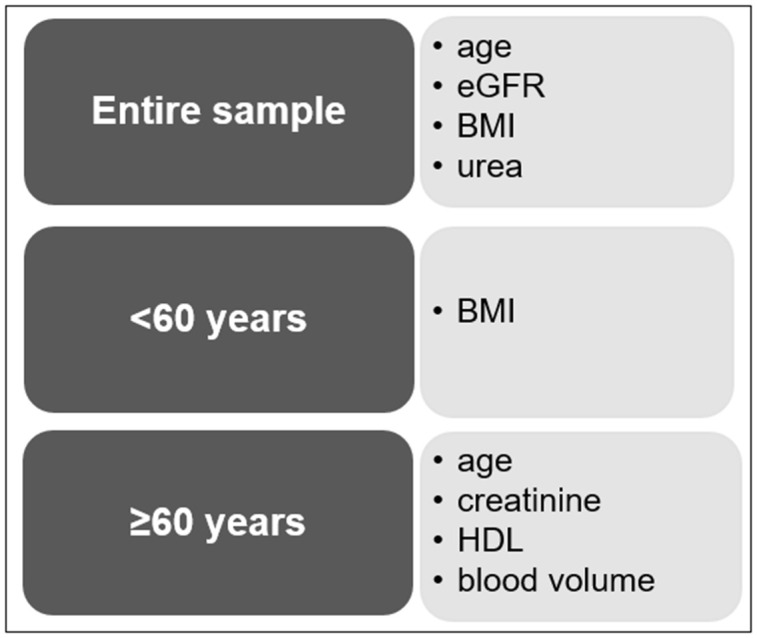
Summary of the most important variables affecting the sNfL concentration.

In individuals of above 60 years regression analysis revealed that age (β=0.394; 95% CI: 0.868, 1.632; p<0.001), creatinine (β=0.376; 95% CI: 13.912, 35.886; p<0.001), blood volume (β=-0.198; 95% CI: -6.797, -1.048; p=0.008) and HDL (β=0.149; 95% CI: 0.01, 0.215; p=0.013) were significantly associated with sNfL. This model explained 37.9% of the variance. BMI (p=0.246) and urea (p=0.435) were unrelated to sNfL in this analysis (for summary see Figure 2).

In individuals below 60 years BMI (β=-0.298; 95% CI: -0.719, -0.144; p=0.013) was the only determinant significantly associated with sNfL. In this model, blood volume (p=0.600), eGFR (p=0.126) and age (p=0.222) were no longer significantly associated with sNFL. Overall, the model explained 17.0% of the variance.

## DISCUSSION

In this study we comprehensively investigated factors influencing sNfL levels and determined their relative importance across different age groups in neurologically inconspicuous individuals.

We show that age increasingly affects sNfL levels, especially in individuals above 60 years. In this age group we found that other variables like renal function and blood volume also factor in, although at a much lower scale. In contrast, in younger individuals (<60 years), we identified BMI as the most important factor influencing sNfL.

sNfL is currently one of the most promising biomarkers indicating neuro-axonal damage in various acute and chronic neurological disorders [5]. However, there are still some limiting factors which hamper its application in clinical routine. One and foremost the already well known increase of sNfL with age and its rise in variability makes it difficult to establish age related cut-off levels to dissect normal from pathological levels [5]. There are a few recent reports showing that sNfL levels may further be influenced by other factors, including blood volume and BMI [6, 7]. Up to now it was completely unclear to which extent these factors impact sNfL levels when analysed combinedly across different age groups. However, the exact knowledge of factors influencing sNfL is inevitable to allow adequate interpretation of sNfL levels in the clinical setting.

When considering the entire cohort, the combined analyses of all included variables showed that age was by far the most relevant factor influencing sNfL concentration, which was increasingly relevant in individuals above the age of 60 years. This is in line with previous findings in neurologically inconspicuous individuals showing that around the age of 60 years sNfL levels profoundly increase in a non-linear manner, which is paralleled by a considerable rise in variability of this marker [5]. A similar non-linear increase in sNfL levels with higher age has been reported in two other recent publications [4, 9]. One can speculate that the rising slope in sNfL increase in the elderly may be caused by concomitant evolving yet clinically silent pathological processes [5]. Interestingly, the increase in sNfL in a normal aging cohort was associated with brain volume loss over time, supporting the notion that other factors beyond normal aging may contribute to sNfL increase in older individuals (>60 years) [5]. The hypothesis of parallel neurodegeneration is corroborated by a study in healthy elderly subclinical Alzheimer’s disease patients revealing an association between plasma NfL and an AD-related biomarker (t-tau) [10], which has been interpreted to reflect the degree of neurodegenerative changes.

In this context it is important to highlight that other factors than neuro-axonal damage may also contribute to an increase in sNfL with higher age, including a reduced cerebrospinal fluid turn over [11], and the increase of the likelihood of coexisting comorbidities with higher age, which may potentially also influence sNfL levels. Although the impact of some of these variables (renal function, blood volume, BMI) has already separately been examined in the past [4–6, 8, 12, 13], a collective analysis identifying the actual relative importance of each confounding factor has so far not been undertaken. Indeed, our combined analytical approach revealed that apart from age, renal function and blood volume also influence sNfL levels in elderly individuals (>60 years), although compared to age at a much lower scale.

Current literature on renal function and sNfL is scarce. An a prior study on the association between renal function and sNfL in diabetic patients found subjects with GFR below 60 having 8-times higher odds of increased sNfL concentrations than those with GFR in normal range [14]. Similarly, sNfL and serum creatinine levels were correlated even after adjusting for age, sex and BMI in healthy elderly (mean age 62.2 years) and diabetic patients (mean age 75.2 years) [8]. The authors of the respective study suggest that sNfL might be cleared by the kidneys, implying that chronic kidney disease should be considered when interpreting sNfL concentrations [14]. Our results point toward the same direction showing that higher sNfL concentration is associated with lower renal function in subjects above 60 years.

Blood volume is a determinant dependent of height and weight. In a prior study, blood volume has been negatively associated with the plasma NfL concentration irrespective of sex and age at sampling in healthy individuals but also in patients suffering from multiple sclerosis [6]. Corroborating these results, we suggest to consider blood volume upon interpreting blood NfL concentration especially in older individuals.

In contrast in younger individuals below 60 years the combined analysis of all included variables revealed that BMI was the most important factor influencing sNfL. It is somehow surprising, that in this group of younger individuals, BMI outweighs age as the most important factor for sNfL. The confounding effect of BMI on sNfL concentration found in our study is in line with previous reports [6, 7]. In a preliminary study, a negative correlation between BMI and sNfL was reported in a healthy cohort from a population-based study even when adjusting for age and sex [6]. Similar results were also found in young anorectic women when compared to recovered and healthy females. Whilst anorectic women had the highest sNfL concentration, recovered women showed a recovery towards the NfL concentration of healthy women [7]. The underlying mechanisms causing the inverse relationship between sNfL and BMI needs to be further determined and its clinical relevance warrants further investigation.

This study has also some limitations, which need to be acknowledged. First, in this study we examined neurologically healthy middle-aged and older individuals. Determinants affecting the sNfL concentration in younger individuals below 30 years needs to be evaluated in future studies. Second, our study may not serve to study the impact of acute and chronic kidney disease on the sNfL concentration, which should be investigated in future studies. Nevertheless, our study provides new insights into the age-related impact of factors influencing sNfL and we believe that it constitutes an important basis to further validate this marker for its use in clinical practice.

In summary we here provide detailed analysis of confounding factors of sNfL in a neurologically inconspicuous cohort. We show that age is by far the most important factor influencing sNfL levels. However, subgroup analyses revealed that this association is mainly driven by individuals above 60 years. In addition to age, renal function and blood volume further influence sNfL levels in elderly people (>60 years), although at a much lower scale. In contrast, applying a combined statistical analysis we found that in younger individuals (<60 years), BMI was the strongest influencing factor of sNfL, even outperforming the variable age in this subgroup.

Our results demonstrate the relative importance of confounding factors of sNfL change with increasing age, which should be considered when interpreting blood levels of this marker in the clinical setting.

## MATERIALS AND METHODS

### Ethics

The study protocol was approved by the ethics committee of the Medical University of Graz, Austria, and written informed consent was obtained from all participants.

### Participants

In this study we analyzed neurologically inconspicuous individuals who participated in the Austrian Stroke Prevention Family Study (ASPS-Fam), an extension of the Austrian Stroke Prevention Study (ASPS) [15, 16]. The ASPS is a prospective single-centre community-based study established in 1991 on the cerebral effects of vascular risk factors in the normal aged population of the city of Graz, Austria. For the ASPS-Fam a total of 419 individuals were included. Between 2006 and 2013 study participants underwent thorough clinical history taking and neurological examination, magnetic resonance imaging (MRI), laboratory evaluation and cognitive testing accompanied by a comprehensive vascular risk factor assessment. Inclusion criteria were no history of previous stroke or dementia and a normal neurologic examination. None of the subjects had suffered a traumatic brain injury. From the 419 individuals included in the ASPS-Fam, blood sampling was available in 371 subjects. Another 44 subjects were excluded due to diagnosis or suspicion of dementia (Mini Mental Status Examination (MMSE) ≤24, n=11), visible brain infarcts on Magnetic Resonance Imaging (MRI) (n=19), a history of stroke (n=9), other diseases (chronic myeloid leukemia) (n=1), missing laboratory data (n=4) or a combination of the above (n=3), leaving 327 subjects to examine confounding factors of sNfL alteration in a normal aging population.

### Demographic, life-style and clinical variables

Overall, the effect of 24 determinants potentially affecting sNfL was assessed. We included demographical factors (age, sex) and life-style related factors (waist-hip-ratio (WHR), body mass index (BMI), alcohol consumption). Laboratory variables of renal and liver function included the eGFR, creatinine, urea, uric acid, and GPT, glutamic oxaloacetic transaminase (GOT), gamma-glutamyl transferase (GGT), alkaline phosphatase (AP). Estimated GFR was calculated as suggested by the National Kidney Foundation estimating GFR [17] from serum creatinine, age, sex and race using the formula *eGFR = 141 x min(SCr/κ, 1)α x max(SCr /κ, 1)-1.209 x 0.993Age x 1.018 [if female] x 1.159 [if Black]* with SCr being standardized serum creatinine (mg/dL), κ = 0.7 for females and 0.9 for males, α = -0.329 for females and -0.411 for males, min indicates the minimum of SCr/κ or 1, max indicates the maximum of SCr/κ or 1 and age in years. Further, cerebrovascular risk factors (hypertension, hypercholesterolemia, diabetes, mean systolic and diastolic blood pressure, cholesterol, low- (HDL) and high-density lipid protein (LDL), triglycerides, glycated hemoglobin (HBA1C)) and blood volume were included. Blood volume was calculated separately for males and females based on weight and height using the formula by Nadler: blood volume = 0.3669 * height^3^ + 0.03219 * weight + 0.6041 for males and blood volume = 0.3561 * height^3^ + 0.03308 * weight + 0.1833 for females as in [18].

### Definition of categorical variables

Alcohol consumption was defined as daily uptake exceeding half a liter beer, a glass of wine or 1cl of high-proof alcohol. Hypertension was defined as use of antihypertensive therapy or systolic blood pressure of 140mmHg or above or diastolic blood pressure of 90mmHg or above [19]. Hypercholesterolemia was considered if the subject was on therapy. Diabetes was considered if the subject either was on therapy, blood sugar level exceeded 126 mg/dl or a positive medical history [19].

### NfL measurement

Serum samples of all participants were collected by venipuncture and processed on the same day of examination between 2006 and 2013 within 2h. After venipuncture, blood tubes remained at room temperature for 30 min and were then centrifuged at room temperature for 10 min at 2000 x g. Serum was then aliquoted in polypropylene tubes and stored at -70° C until analysis of NfL.

All serum samples were analyzed at the University Hospital Basel, Switzerland as previously described [5]. In brief, serum NfL levels were determined by single molecule array (Simoa) assay using the capture monoclonal antibody (mAB) 47:3 (initial dilution 0.3 mg/mL; Art. No. 27016) and the biotinylated detector mAB 2:1 (0.1 μg/mL; Art. No. 27018) [20]. The mean coefficients of variation (CVs) of duplicate determinations for concentration were 8.5% (9.5 pg mL^-1^, sample 1), 5.4% (23.2 pg mL^-1^, sample 2) and 7.8% (98.5 pg mL^-1^, sample 3). Interassay CVs for serum were 7.8% (sample 1), 8.3% (sample 2) and 4.9% (sample 3) [5].

### Statistical analyses

Statistical analysis was performed using SPSS (version 26, SPSS Inc., Chicago, IL, USA) and the statistical software R (version 4.0.2; R: a language and environment for statistical computing, Vienna, Austria). A two-sided p-value <0.05 was considered to be statistically significant. Explorative data analysis with extreme scores on the upper and lower ends of the score distributions were used for the identification of outliers. A score was considered an outlier if it exceeded 1.5 x interquartile range (IQR) of a variable.

The entire cohort was also stratified by age in the age groups <60 years (age range: 38.5-59.9, n=99) and 60 years and above (age range: 60.1-84.3, n=228) equivalent to [5]. This stratification was chosen due to the non-linear relationship between sNfL and age showing increasing sNfL concentrations above the age of 60 potentially indicating a subclinical pathological process.

Spearman correlations were used to identify determinants significantly correlated with sNfL in either the entire cohort or the stratified samples. With respect to categorical variables, Mann-Whitney-U-Tests were used to identify differences in the sNfL level. Significant variables were used in further analyses. Variables with non-significant association with sNfL were excluded from further analyses.

To determine the conditional importance of factors influencing sNfL, we calculated random forest regressions. Random forest assesses the explanatory power of each individual variable while at the same time accounting for all other variables. Using the R package ‘party’ (version 1.3-5 [21]) 1001 inference trees with unbiased variable selection were calculated using 5 randomly selected variables for each split (unbiased resampling scheme). From these trees conditional permutation importance was calculated, using the “mean decrease in accuracy” principle as importance measure for each variable together with a 95% confidence interval from 400 repetitions. This approach was chosen (1) to account for the intercorrelation between predictor variables (multicollinearity), (2) to uncover variables that are non-linearly related to sNfL and, ultimately, (3) to identify the variables with highest conditional variable importance. Random forest regressions were calculated in the entire cohort and in the age-stratified samples.

Determinants for the sNfL level having the lower limit of the confidence interval >0 were then used in multiple linear regressions including bootstrapping with replacement (10.000 samples, bias corrected and accelerated (BCa-method), 95% confidence interval) to determine significant associations with sNfL. In case of correlation of variables, one was excluded from the multiple regression analysis due to multicollinearity (variance inflation factor, VIF>5). Bootstrapping was chosen for deriving more robust estimates of the standard error and confidence intervals. Lower and upper 95% confidence intervals from the bootstrapped coefficients were estimated to determine significance. If the 95% confidence interval (CI) of the indirect effect does not contain 0, a significant effect is probable.

### Data availability

Data sets generated and/or analyzed during the study are available from the corresponding author upon reasonable request.
